# Supplementary material for: Visualization of translation and protein biogenesis at the ER membrane
Source: Nature. 2023 Jan 25;614(7946):160–7. doi: 10.1038/s41586-022-05638-5 (PMC9892003; doi:10.1038/s41586-022-05638-5)
Supplement: Supplementary file 1 — This file contains Supplementary Figs. 1–7 and Supplementary Tables 1 and 2. [file 41586_2022_5638_MOESM1_ESM.docx]

Supplementary Information for

**Visualization of translation and protein biogenesis at the ER membrane**

Max Gemmer^1^, Marten L. Chaillet^1^, Joyce van Loenhout^1^, Rodrigo Cuevas Arenas^1^, Dimitrios Vismpas^1^, Mariska Gröllers-Mulderij^1^, Fujiet A. Koh^2^, Pascal Albanese^3,4^, Richard Scheltema^3,4^, Stuart C. Howes^1^, Abhay Kotecha^2^, Juliette Fedry^1,5^, Friedrich Förster^1,5^

1: Structural Biochemistry, Bijvoet Center for Biomolecular Research, Utrecht University, 3584 CG Utrecht, The Netherlands

2: Thermo Fisher Scientific, Eindhoven, The Netherlands

3: Biomolecular Mass Spectrometry and Proteomics Group, Utrecht Institute for Pharmaceutical Sciences, Utrecht University, 3584 CH Utrecht, The Netherlands

4: Netherlands Proteomics Center, Utrecht University, Utrecht University, 3584 CH Utrecht, The Netherlands

5: Correspondence to: j.m.m.fedry@uu.nl and f.g.forster@uu.nl

Table of Contents

[Supplementary Figures 3](#_Toc120287872)

[Supplementary Tables 12](#_Toc120287873)

[Supplementary Video Captions 23](#_Toc120287874)

# Supplementary Figures


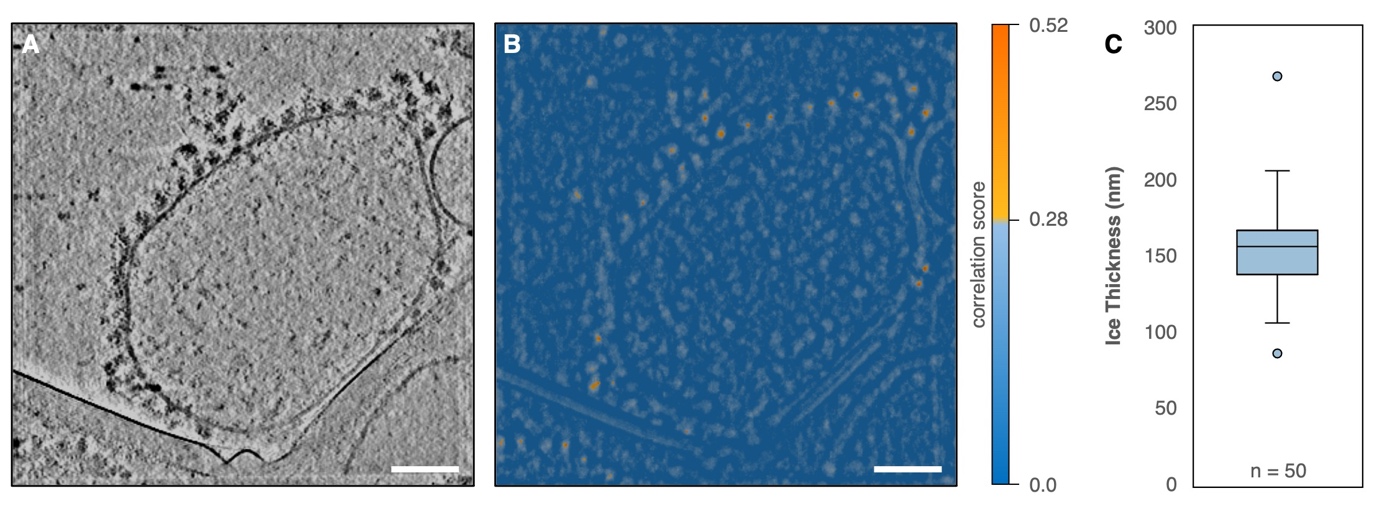


**Supplementary Fig. 1: Cryo-tomograms of ER-derived vesicles from HEK-293 cells.** (A) 2D slice (thickness 2.0 nm) of a representative tomogram. The scalebar corresponds to 100 nm. (B) PyTom correlation scores resulting from template matching ^69^. Particles with scores of 0.28-0.56 were selected for subsequent subtomogram analysis. Scores are color-coded as indicated. (C) Distribution of ice thickness of n = 50 randomly selected tomograms. Median thickness (156 nm) is indicated, the box defines the lower (138 nm) and upper (167 nm) quartile, whiskers define the minimum (106 nm) and maximum (206 nm) thickness. Outliers are indicated.


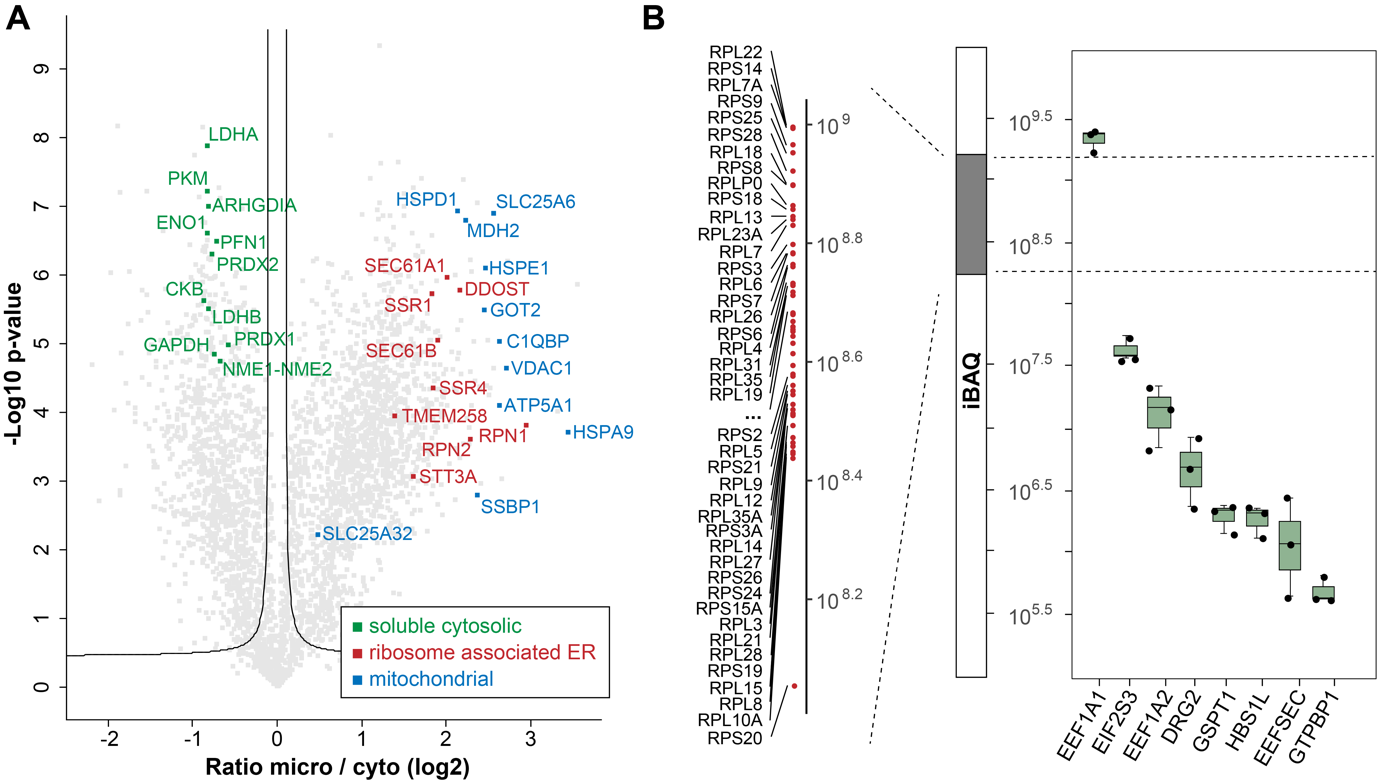


**Supplementary Fig. 2: Proteomics characterization of microsome preparation.** (A) Enrichment of ribosome-associated ER and mitochondrial proteins and depletion of cytosolic proteins when comparing microsome enriched samples to cytosolic supernatant. The y-axis of the volcano plot was made using a two-sided T-test, where the false positive rate was controlled to 5% based on 250 randomizations of the data. (B) Dynamic range of the detected absolute abundances for the measured proteome (central bar labeled with intensity-based quantification, iBAQ). Ribosomal proteins occupy a narrow band of absolute abundances (left bar). Among ribosome-binding GTPases, eEF1a the most abundant (right boxplot). Data points show n=3 technical replicates of 1 experiment. Median of logarithmic of IBAQ values is indicated, the box represents lower and upper quartile, whiskers represent minimum and maximum.


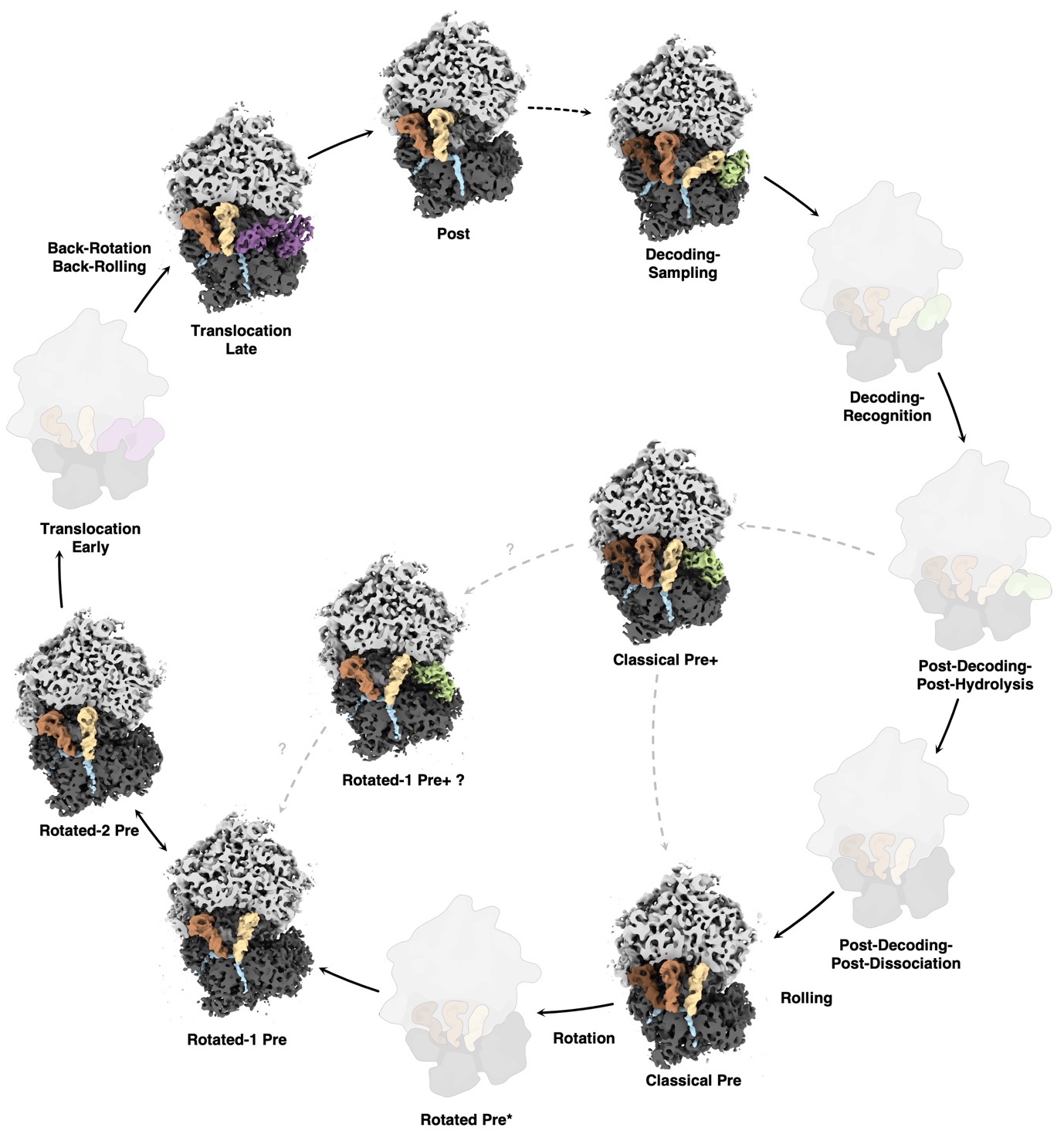


**Supplementary Fig. 3: Observed active intermediates in the context of the complete model of human elongation cycle.** Different from Figure 1D all observed ribosomal states are positioned in a model recapitulating structurally characterized states beyond the most abundant ones detected in our study. States that are not detected are grayed out. The solid arrows indicate the cycle from ^21^, while the transitions to states only detected in our study are broken lines. The Rotated-1 Pre+ state had not been assigned in Fig. 1D due to its missing support from *in situ* data.


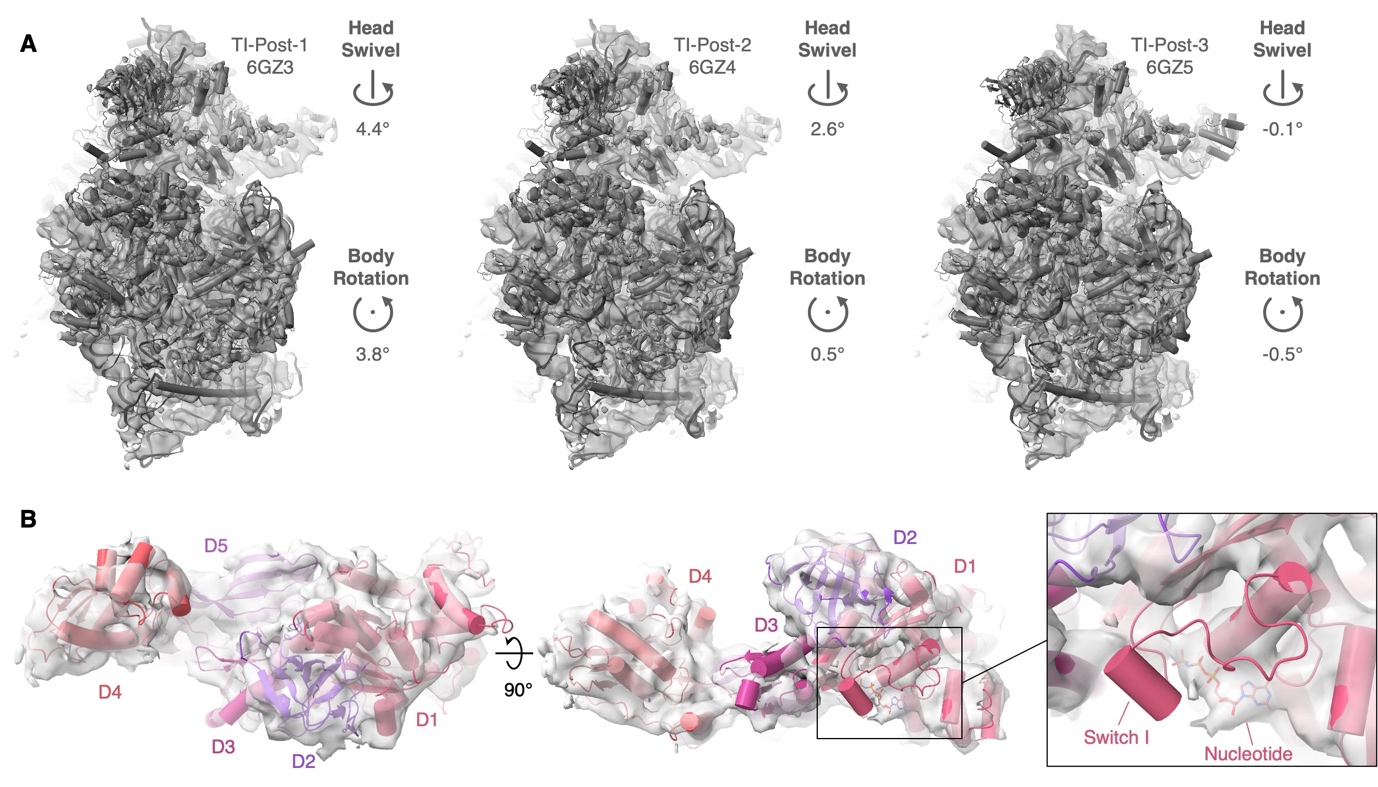
 **Supplementary Fig. 4: Identification of elongation factor-bound ribosomal translocation intermediate states.** (A) Large ribosomal subunits of the TI (translocation intermediate)-Post-1 (6GZ3), TI-Post-2 (6GZ4) and TI-Post-3 (6GZ5) states were fitted into our reconstruction of the ‘translocation’ intermediate from Fig. 1A and the structure of the small ribosomal subunit was superposed onto our reconstruction. Rotational differences of the SSU head and body with respect to our reconstruction are indicated. (B) Domains of eEF2 (6GZ5) fitted into the translocation intermediate. Domains D1, D2, D4 and D5 fit well into the reconstruction, domain D3 is positioned slightly differently. The switch I loop is disordered in our reconstruction indicating a GDP-bound state.


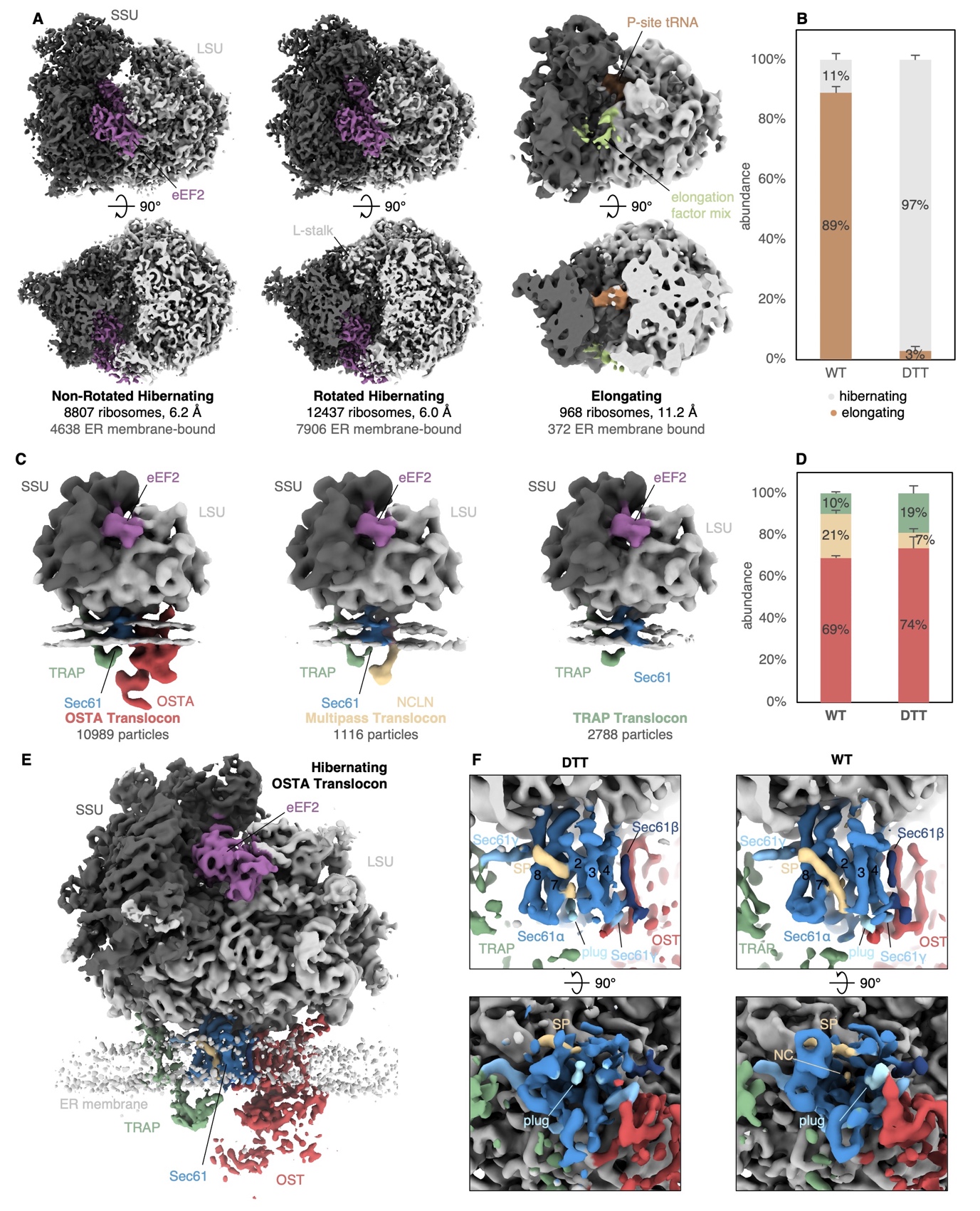


**Supplementary Fig. 5: Cryo-ET analysis of ER membrane-bound ribosomes obtained from stressed cells.** (A) Side views (top row) and top views (bottom row) of intermediate states of soluble and ER membrane-associated ribosomes under condition of oxidative stress. (B) Abundance of elongating and hibernating ER membrane-bound ribosomes in WT and DTT-treated vesicles. n(WT) = 132,371 particles in 869 tomograms from 1 experiment, n(DTT) = 26,512 particles in 212 (174 and 38) tomograms from 2 independent experiments. Stacked columns show the modelled mean with the 95% confidence interval as error bars. (C) Different populations of ER translocons from DTT-treated microsomes. (D) Distribution of ER translocon populations in WT and DTT-treated sample (C). n(WT) = 132,371 particles in 869 tomograms from 1 experiment, n(DTT) = 26,512 particles in 212 (174 and 38) tomograms from 2 independent experiments. Stacked columns show the modelled mean with the 95% confidence interval as error bars. (E) Structure of the hibernating ribosome-bound OSTA-translocon complex from DTT-treated samples at an overall resolution of 6.4 Å. (F) Side view (top row) and view from the ER lumen (bottom row) of the active WT (left) and inactive DTT-treated (right) OSTA-translocon. Both reconstructions were filtered to a resolution of 8 Å. SP: signal peptide, NC: nascent chain.


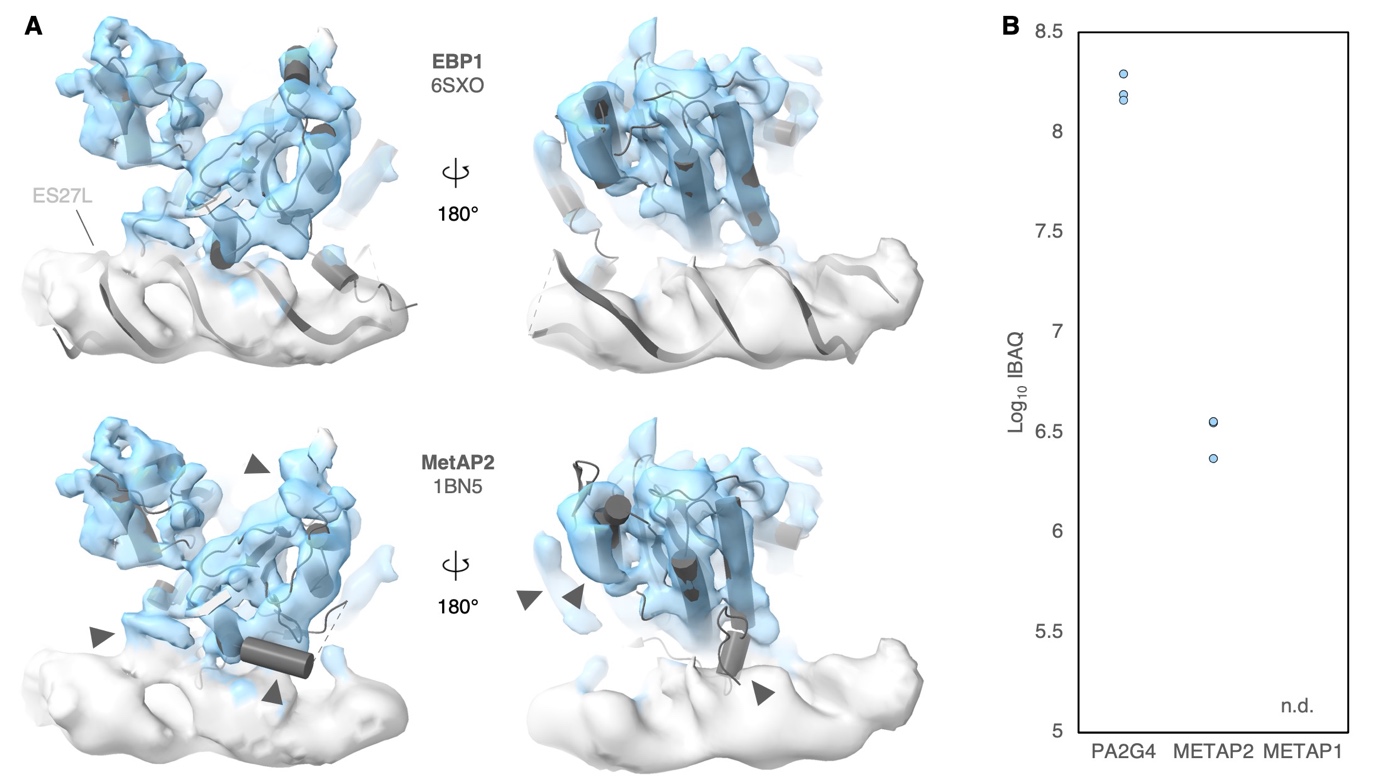
**Supplementary Fig. 6: Identification of ribosome-associated EBP1.** (A) Structures of EBP1 (6SXO, SPA structure) and MetAP2 (1BN5, crystal structure) fitted into our segmented reconstruction of soluble ribosomes. Arrowheads indicate structural differences between EBP1 and MetAP2 that are not explained by the reconstruction. (B) Abundance of the structurally related proteins EBP1 (PA2G4), MetAP1 and MetAP2 in our ER-vesicle preparation determined by mass spectrometry. n.d. – not determined. Data points show n=3 technical replicates of 1 experiment.


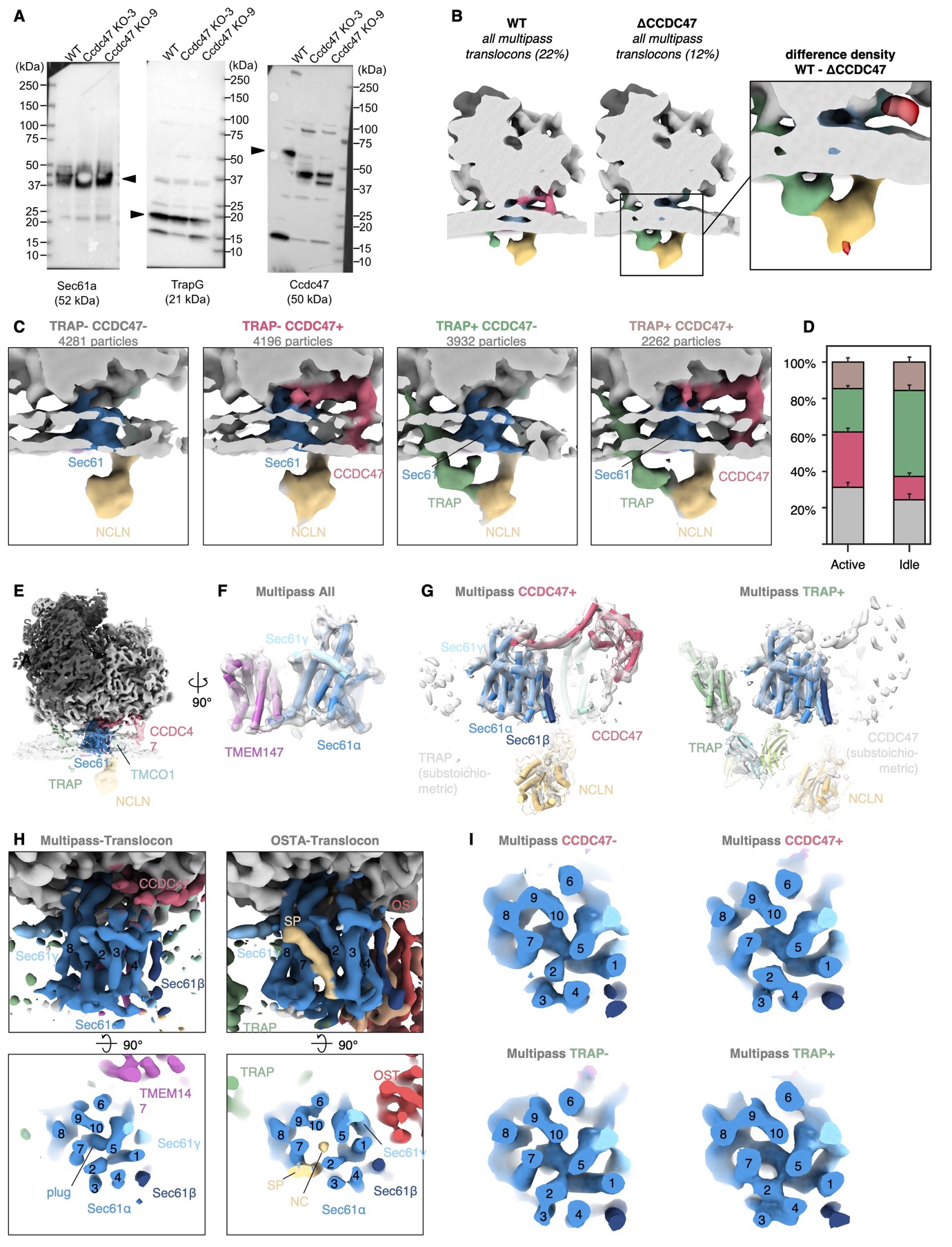


**Supplementary Fig. 7: Reconstructions of multipass translocon populations.** (A) Western blots of ER-derived vesicles prepared from HEK293F WT and ΔCCDC47 cells. Samples of WT and knock-out (KO) clones 3 and 9 in different lanes derive from the same experiment. The membrane was cut vertically after blotting and its fragments were processed in parallel. Sec61α (Sec61a) and TRAPγ (TrapG) were used as ER marker. The depicted Western blot is a representative of two independent experiments. (B) Comparison between the multipass translocon population from WT and ΔCCDC47. The difference density is displayed at 2σ (red). n(ΔCCDC47) = 555 particles in 60 tomograms from 1 experiment (C) Multipass translocon populations obtained by classification focused on the ER luminal densities of TRAP and NCLN or on CCDC47. (D) Quantification of multipass translocon populations associated with elongating or hibernating ribosomes. Color-code as in (C). n = 14781 particles in 869 tomograms from 1 experiment. (E) Overall structure of the entire multipass translocon-associated ribosome filtered to 8 Å (opaque) and 20 Å (transparent) resolution. Densities of TRAP, NCLN, CCDC47 are highly fragmented due to flexibility or compositional heterogeneity. (F) Close-up view of Sec61 and TMEM147 (PDB: 6W6L) fitted into the segmented reconstruction from (E). (G) Components of the multipass translocon (PDB: 6W6L) fitted into the segmented densities of CCDC47-containing or TRAP-containing populations. Densities that are distant from the ribosome are fragmented due to flexibility. (H) Close-up views of Sec61 from the entire multipass-translocon population (left) compared to the OSTA-translocon (right). Numbering of TMHs of Sec61α is indicated. SP– signal peptide, NC – nascent chain. (I) Segmented density of Sec61 shown as in (H) of reconstructions from populations lacking or containing CCDC47 and lacking or containing TRAP.

# Supplementary Tables

**Supplementary Table 1: Cryo-EM data collection, refinement and validation statistics of ribosome and translocon classes**

|  | #1  Decoding-Sampling State  (EMDB-15871) | #2  Classical Pre+ State  (EMDB-15872) | #3  Classical Pre State  (EMDB-15873) | #4  Rotated-1 Pre+ State  (EMDB-15874) | #5  Rotated-1 Pre State  (EMDB-15875) |
| --- | --- | --- | --- | --- | --- |
| **Data collection and processing** |  |  |  |  |  |
| Magnification | 79000 | 79000 | 79000 | 79000 | 79000 |
| Voltage (kV) | 200 | 200 | 200 | 200 | 200 |
| Electron exposure (e–/Å^2^) | <80 | <80 | <80 | <80 | <80 |
| Defocus range (μm) | 3 | 3 | 3 | 3 | 3 |
| Pixel size (Å) | 1.724 | 1.724 | 1.724 | 1.724 | 1.724 |
| Symmetry imposed | - | - | - | - | - |
| Initial particle images (no.) | 134350 | 134350 | 134350 | 134350 | 134350 |
| Final particle images (no.) | 26980 | 40035 | 3884 | 4196 | 5300 |
| Map resolution (Å)  FSC threshold | 4.8  0.143 | 4.6  0.143 | 8.0  0.143 | 6.7  0.143 | 6.1  0.143 |
| Map resolution range (Å) | 3.6-51.7 | 3.6-34.5 | 4.3-51.7 | 4.3-51.7 | 4.1-51.7 |
|  |  |  |  |  |  |
| **Refinement** |  |  |  |  |  |
| Initial model used (PDB code) |  |  |  |  |  |
| Model resolution (Å)  FSC threshold |  |  |  |  |  |
| Model resolution range (Å) |  |  |  |  |  |
| Map sharpening *B* factor (Å^2^) |  |  |  |  |  |
| Model composition  Non-hydrogen atoms  Protein residues  Ligands |  |  |  |  |  |
| *B* factors (Å^2^)  Protein  Ligand |  |  |  |  |  |
| R.m.s. deviations  Bond lengths (Å)  Bond angles (°) |  |  |  |  |  |
| Validation  MolProbity score  Clashscore  Poor rotamers (%) |  |  |  |  |  |
| Ramachandran plot  Favored (%)  Allowed (%)  Disallowed (%) |  |  |  |  |  |

|  | #6  Rotated-2 State  (EMDB-15876) | #7  Translocation State  (EMDB-15877) | #8  Post State  (EMDB-15878) | #9  Non-Rotated Hibernating  (EMDB-15879) | #10  Rotated Hibernating  (EMDB-15880) |
| --- | --- | --- | --- | --- | --- |
| **Data collection and processing** |  |  |  |  |  |
| Magnification | 79000 | 79000 | 79000 | 79000 | 79000 |
| Voltage (kV) | 200 | 200 | 200 | 200 | 200 |
| Electron exposure (e–/Å^2^) | <80 | <80 | <80 | <80 | <80 |
| Defocus range (μm) | 3 | 3 | 3 | 3 | 3 |
| Pixel size (Å) | 1.724 | 1.724 | 1.724 | 1.724 | 1.724 |
| Symmetry imposed | - | - | - | - | - |
| Initial particle images (no.) | 134350 | 134350 | 134350 | 134350 | 134350 |
| Final particle images (no.) | 20560 | 5789 | 13511 | 7565 | 4551 |
| Map resolution (Å)  FSC threshold | 4.8  0.143 | 6.8  0.143 | 5.9  0.143 | 6.5  0.143 | 6.7  0.143 |
| Map resolution range (Å) | 3.6-51.7 | 3.6-51.7 | 3.6-51.7 | 4.3-51.7 | 4.1-51.7 |
|  |  |  |  |  |  |
| **Refinement** |  |  |  |  |  |
| Initial model used (PDB code) |  |  |  |  |  |
| Model resolution (Å)  FSC threshold |  |  |  |  |  |
| Model resolution range (Å) |  |  |  |  |  |
| Map sharpening *B* factor (Å^2^) |  |  |  |  |  |
| Model composition  Non-hydrogen atoms  Protein residues  Ligands |  |  |  |  |  |
| *B* factors (Å^2^)  Protein  Ligand |  |  |  |  |  |
| R.m.s. deviations  Bond lengths (Å)  Bond angles (°) |  |  |  |  |  |
| Validation  MolProbity score  Clashscore  Poor rotamers (%) |  |  |  |  |  |
| Ramachandran plot  Favored (%)  Allowed (%)  Disallowed (%) |  |  |  |  |  |

|  | #11  Ribosome-Sec61-TRAP-OSTA  (EMDB-15884) | #12  Ribosome-Sec61-TRAP  (EMDB-15885) | #13  Ribosome-Sec61-TRAP-Multipass  (EMDB-15886) | #14  Ribosome-Sec61-Multipass  (EMDB-15887) | #15  Ribosome-EBP1  (EMDB-15888) |
| --- | --- | --- | --- | --- | --- |
| **Data collection and processing** |  |  |  |  |  |
| Magnification | 79000 | 79000 | 79000 | 79000 | 79000 |
| Voltage (kV) | 200 | 200 | 200 | 200 | 200 |
| Electron exposure (e–/Å^2^) | <80 | <80 | <80 | <80 | <80 |
| Defocus range (μm) | 3 | 3 | 3 | 3 | 3 |
| Pixel size (Å) | 1.724 | 1.724 | 1.724 | 1.724 | 1.724 |
| Symmetry imposed | - | - | - | - | - |
| Initial particle images (no.) | 134350 | 134350 | 134350 | 134350 | 134350 |
| Final particle images (no.) | 42215 | 6725 | 6194 | 8477 | 24577 |
| Map resolution (Å)  FSC threshold | 4.5  0.143 | 6.5  0.143 | 7.5  0.143 | 6.7  0.143 | 5.0  0.143 |
| Map resolution range (Å) | 3.6-51.7 | 4.1-51.7 | 4.5-51.7 | 4.3-51.7 | 3.8-51.7 |
|  |  |  |  |  |  |
| **Refinement** |  |  |  |  |  |
| Initial model used (PDB code) |  |  |  |  |  |
| Model resolution (Å)  FSC threshold |  |  |  |  |  |
| Model resolution range (Å) |  |  |  |  |  |
| Map sharpening *B* factor (Å^2^) |  |  |  |  |  |
| Model composition  Non-hydrogen atoms  Protein residues  Ligands |  |  |  |  |  |
| *B* factors (Å^2^)  Protein  Ligand |  |  |  |  |  |
| R.m.s. deviations  Bond lengths (Å)  Bond angles (°) |  |  |  |  |  |
| Validation  MolProbity score  Clashscore  Poor rotamers (%) |  |  |  |  |  |
| Ramachandran plot  Favored (%)  Allowed (%)  Disallowed (%) |  |  |  |  |  |

|  | #16  Idle Ribosome-Sec61-TRAP-OSTA  (EMDB-15889) | #17  Sec61-TRAP-OSTA- Translocon (all)  (EMDB-15870)  (PDB 8B6L) | #18  Sec61-TRAP-OSTA- Translocon  (EMDB-15890) | #19  Sec61-TRAP-OSTA-L1-Translocon  (EMDB-15891) | #20  Sec61-TRAP-OSTA-L2-Translocon  (EMDB-15892) |
| --- | --- | --- | --- | --- | --- |
| **Data collection and processing** |  |  |  |  |  |
| Magnification | 79000 | 79000 | 79000 | 79000 | 79000 |
| Voltage (kV) | 200 | 200 | 200 | 200 | 200 |
| Electron exposure (e–/Å^2^) | <80 | <80 | <80 | <80 | <80 |
| Defocus range (μm) | 3 | 3 | 3 | 3 | 3 |
| Pixel size (Å) | 1.724 | 1.724 | 1.724 | 1.724 | 1.724 |
| Symmetry imposed | - | - | - | - | - |
| Initial particle images (no.) | 134350 | 134350 | 134350 | 134350 | 134350 |
| Final particle images (no.) | 9163 | 42215 | 4555 | 14541 | 14991 |
| Map resolution (Å)  FSC threshold | 6.4  0.143 | 7.6  0.143 | 9.3  0.143 | 8.2  0.143 | 8.3  0.143 |
| Map resolution range (Å) | 4.3-51.7 | 5.4-51.7 | 6.5-51.7 | 5.4-51.7 | 5.7-51.7 |
|  |  |  |  |  |  |
| **Refinement** |  |  |  |  |  |
| Initial model used (PDB code) |  | AF and Colab models |  |  |  |
| Model resolution (Å)  FSC threshold |  | 6.0  0.5 |  |  |  |
| Model resolution range (Å) |  |  |  |  |  |
| Map sharpening *B* factor (Å^2^) |  |  |  |  |  |
| Model composition  Non-hydrogen atoms  Protein residues  Ligands (RNA) |  | 29611  3798  0 |  |  |  |
| *B* factors (Å^2^)  Protein  Ligand (RNA) |  | 328.57  - |  |  |  |
| R.m.s. deviations  Bond lengths (Å)  Bond angles (°) |  | 0.003  0.594 |  |  |  |
| Validation  MolProbity score  Clashscore  Poor rotamers (%) |  | 1.26  2.19  0% |  |  |  |
| Ramachandran plot  Favored (%)  Allowed (%)  Disallowed (%) |  | 96.11%  3.81%  0.08% |  |  |  |
|  |  |  |  |  |  |

|  | #21  SPA reconstruction Classical Pre+ State  (EMDB-15893)  (PDB 8B6Z) |
| --- | --- |
| **Data collection and processing** |  |
| Magnification |  |
| Voltage (kV) | 300 |
| Electron exposure (e–/Å^2^) | 40 |
| Defocus range (μm) |  |
| Pixel size (Å) | 0.729 |
| Symmetry imposed | - |
| Initial particle images (no.) | 66041 |
| Final particle images (no.) | 19046 |
| Map resolution (Å)  FSC threshold | 2.9  0.143 |
| Map resolution range (Å) | 2.3-22.7 |
|  |  |
| **Refinement** |  |
| Initial model used (PDB code) | 4C0S |
| Model resolution (Å)  FSC threshold | 3.2  0.5 |
| Model resolution range (Å) | - |
| Map sharpening *B* factor (Å^2^) | 0 |
| Model composition  Non-hydrogen atoms  Protein residues  Ligands (RNA) | 3581  442  8 |
| *B* factors (Å^2^)  Protein  Ligand (RNA) | 47.78  51.17 |
| R.m.s. deviations  Bond lengths (Å)  Bond angles (°) | 0.002  0.505 |
| Validation  MolProbity score  Clashscore  Poor rotamers (%) | 0.70  0.59  0.27% |
| Ramachandran plot  Favored (%)  Allowed (%)  Disallowed (%) | 98.64%  1.36%  0% |
|  |  |

**Supplementary Table 2: Most abundant proteins determined by mass-spectrometry analysis of ER-derived microsomes and its supernatant from HEK293 cells (full dataset deposited on PRIDE database with identifier PXD035475).**

| **Protein IDs** | **Gene names** | **iBAQ supernatent** | **iBAQ microsomes** |
| --- | --- | --- | --- |
| P63261 | ACTG1 | 4.8E+09 | 8.39E+09 |
| A0A0G2JIW1 | HSPA1B | 7.3E+09 | 3.46E+09 |
| P68104 | EEF1A1 | 3.5E+09 | 2.47E+09 |
| P10809 | HSPD1 | 1.2E+07 | 4.96E+09 |
| P62937 | PPIA | 3.2E+09 | 1.36E+09 |
| Q8N257 | HIST3H2BB | 1.3E+08 | 4.57E+09 |
| P06733 | ENO1 | 3.6E+09 | 6.19E+08 |
| P61604 | HSPE1 | 1.3E+07 | 4.26E+09 |
| K7ERI7 | RPL22 | 6.4E+08 | 9.87E+08 |
| P68363 | TUBA1B | 2.7E+09 | 5.48E+08 |
| A0A5F9ZHM4 | LDHB | 2.6E+09 | 4.39E+08 |
| P46781 | RPS9 | 4.8E+08 | 9.25E+08 |
| P07900 | HSP90AA1 | 2.5E+09 | 4.31E+08 |
| Q5JR95 | RPS8 | 5.8E+08 | 7.9E+08 |
| Q06830 | PRDX1 | 1.9E+09 | 5.66E+08 |
| P62263 | RPS14 | 5.8E+08 | 9.9E+08 |
| P68371 | TUBB4B | 2.2E+09 | 4.16E+08 |
| P04406 | GAPDH | 1.7E+09 | 3.83E+08 |
| P62424 | RPL7A | 3.7E+08 | 9.85E+08 |
| P14625 | HSP90B1 | 2.6E+08 | 2.17E+09 |
| P23396 | RPS3 | 4.7E+08 | 6.28E+08 |
| Q71DI3 | HIST2H3A | 0 | 2.35E+09 |
| G3V203 | RPL18 | 2.1E+08 | 7.91E+08 |
| C9J9K3 | RPSA | 4.2E+08 | 5.48E+08 |
| P62701 | RPS4X | 4.8E+08 | 6.59E+08 |
| Q71UI9 | H2AFV | 4.2E+07 | 2.1E+09 |
| P05388 | RPLP0 | 4.2E+08 | 7.3E+08 |
| P18124 | RPL7 | 2.7E+08 | 6.76E+08 |
| P26641 | EEF1G | 1.2E+09 | 6.97E+08 |
| Q02878 | RPL6 | 3.6E+08 | 6.08E+08 |
| P62857 | RPS28 | 3.6E+08 | 8.35E+08 |
| P08670 | VIM | 1.2E+09 | 7.93E+08 |
| M0QZC5 | RPS11 | 5E+08 | 5.15E+08 |
| P07737 | PFN1 | 1.6E+09 | 2.8E+08 |
| P62899 | RPL31 | 2.5E+08 | 5.4E+08 |
| P62269 | RPS18 | 2.4E+08 | 7.19E+08 |
| Q16777 | HIST2H2AC | 1.5E+07 | 1.73E+09 |
| P62753 | RPS6 | 2.8E+08 | 5.76E+08 |
| P11021 | HSPA5 | 5.5E+07 | 1.65E+09 |
| Q5VVC8 | RPL11 | 2.8E+08 | 4.79E+08 |
| P62081 | RPS7 | 3.5E+08 | 6.06E+08 |
| Q07021 | C1QBP | 1950200 | 1.68E+09 |
| P62244 | RPS15A | 3.1E+08 | 3.23E+08 |
| P36578 | RPL4 | 2.8E+08 | 5.39E+08 |
| C9JXB8 | RPL24 | 2.3E+08 | 4.48E+08 |
| P62851 | RPS25 | 1.1E+08 | 8.96E+08 |
| P12277 | CKB | 1.4E+09 | 2.15E+08 |
| P16403 | HIST1H1C | 4E+07 | 1.46E+09 |
| Q04837 | SSBP1 | 0 | 1.6E+09 |
| P46776 | RPL27A | 2.6E+08 | 4.4E+08 |
| B5MDF5 | RAN | 1.1E+09 | 3.54E+08 |
| P23284 | PPIB | 3.3E+07 | 1.44E+09 |
| Q15084 | PDIA6 | 4.7E+07 | 1.45E+09 |
| E7ETK0 | RPS24 | 2.6E+08 | 3.25E+08 |
| P62249 | RPS16 | 2.7E+08 | 4.26E+08 |
| P25705 | ATP5A1 | 0 | 1.49E+09 |
| P62277 | RPS13 | 2.4E+08 | 4.8E+08 |
| P06576 | ATP5B | 0 | 1.44E+09 |
| P26373 | RPL13 | 1.6E+08 | 7E+08 |
| E9PK54 | HSPA8 | 8.7E+08 | 4.36E+08 |
| Q59GN2 | RPL39P5 | 1.7E+08 | 8.66E+08 |
| P21796 | VDAC1 | 0 | 1.42E+09 |
| B8ZZQ6 | PTMA | 1.2E+09 | 1.33E+08 |
| P35232 | PHB | 0 | 1.42E+09 |
| A0A2R8Y6J3 | RPL5 | 2.7E+08 | 3.93E+08 |
| P12236 | SLC25A6 | 0 | 1.39E+09 |
| P40429 | RPL13A | 2.3E+08 | 4.81E+08 |
| P08238 | HSP90AB1 | 1.1E+09 | 1.97E+08 |
| P27797 | CALR | 7.8E+07 | 1.24E+09 |
| P61956 | SUMO2 | 6.3E+08 | 6.46E+08 |
| P46778 | RPL21 | 2.7E+08 | 2.97E+08 |
| P63244 | GNB2L1 | 2.6E+08 | 3.6E+08 |
| F8VZJ2 | NACA | 7.1E+08 | 3.43E+08 |
| P13639 | EEF2 | 8.3E+08 | 2.39E+08 |
| P05387 | RPLP2 | 2.5E+08 | 4.79E+08 |
| P42677 | RPS27 | 2.4E+08 | 4.66E+08 |
| P25398 | RPS12 | 2.2E+08 | 4.28E+08 |
| P0CG48 | UBC | 5.7E+08 | 5.31E+08 |
| P62805 | HIST1H4A | 0 | 1.23E+09 |
| J3KPX7 | PHB2 | 0 | 1.24E+09 |
| P60174 | TPI1 | 1E+09 | 1.57E+08 |
| P60842 | EIF4A1 | 6.6E+08 | 3.27E+08 |
| D6RAN4 | RPL9 | 2.8E+08 | 3.77E+08 |
| P23528 | CFL1 | 7.1E+08 | 5.13E+08 |
| H0Y8D1 | PRSS1 | 0 | 2.51E+08 |
| J3QRI7 | RPL26 | 1.2E+08 | 5.81E+08 |
| P62829 | RPL23 | 2.1E+08 | 4.45E+08 |
| P07437 | TUBB | 9.9E+08 | 1.99E+08 |
| P38646 | HSPA9 | 0 | 1.21E+09 |
| E7EQV9 | RPL15 | 1.3E+08 | 2.81E+08 |
| E7EPB3 | RPL14 | 1.1E+08 | 3.38E+08 |
| P15880 | RPS2 | 2E+08 | 4.11E+08 |
| A0A3B3ITT5 | RPL29 | 1.7E+08 | 4.78E+08 |
| P61353 | RPL27 | 1.4E+08 | 3.31E+08 |
| P10599 | TXN | 9.5E+08 | 1.51E+08 |
| P13667 | PDIA4 | 3.2E+07 | 1.1E+09 |
| H7BY10 | RPL23A | 8.6E+07 | 6.92E+08 |
| P30050 | RPL12 | 1.5E+08 | 3.63E+08 |
| P00338 | LDHA | 9.6E+08 | 1.53E+08 |
| P39023 | RPL3 | 2E+08 | 3.11E+08 |
| P61247 | RPS3A | 1.8E+08 | 3.51E+08 |
| P62917 | RPL8 | 1.3E+08 | 2.79E+08 |
| P40926 | MDH2 | 1282100 | 1.09E+09 |
| Q8WVC2 | RPS21 | 2.3E+08 | 3.8E+08 |
| P18077 | RPL35A | 1.9E+08 | 3.56E+08 |
| P84098 | RPL19 | 1.1E+08 | 5.22E+08 |
| P53999 | SUB1 | 5.3E+08 | 4.42E+08 |
| P07237 | P4HB | 2.9E+07 | 9.45E+08 |
| P30101 | PDIA3 | 1.7E+07 | 9.46E+08 |
| P14618 | PKM | 8.1E+08 | 1.01E+08 |
| P51149 | RAB7A | 6.2E+07 | 8.66E+08 |
| P17066 | HSPA6 | 5.5E+08 | 3.24E+08 |
| K7ELL7 | PRKCSH | 4.9E+07 | 8.59E+08 |
| P16989 | YBX3 | 2E+08 | 2.94E+08 |
| H0YMF4 | RPL28 | 1.7E+08 | 2.91E+08 |
| Q562R1 | ACTBL2 | 9.2E+08 | 0 |
| P39019 | RPS19 | 1.7E+08 | 2.87E+08 |
| Q32Q12 | NME1-NME2 | 7.3E+08 | 1.6E+08 |
| P22626 | HNRNPA2B1 | 1.7E+08 | 4.89E+08 |
| P62906 | RPL10A | 1.2E+08 | 2.74E+08 |
| P11142 | HSPA8 | 5.6E+08 | 2.79E+08 |
| P09211 | GSTP1 | 7.2E+08 | 1.3E+08 |
| P27824 | CANX | 8759400 | 8.16E+08 |
| Q8NC51 | SERBP1 | 1.3E+08 | 1.8E+08 |
| P45880 | VDAC2 | 0 | 8.36E+08 |
| P19338 | NCL | 4.2E+08 | 3.85E+08 |
| H0Y8G5 | HNRNPD | 2.5E+08 | 2.5E+08 |
| P38117 | ETFB | 0 | 8E+08 |
| P14174 | MIF | 6.6E+08 | 1.29E+08 |
| Q9UQ80 | PA2G4 | 2E+08 | 1.73E+08 |
| E9PR30 | FAU | 9.3E+07 | 4.16E+08 |
| Q00688 | FKBP3 | 1.3E+08 | 1.94E+08 |
| P00403 | MT-CO2 | 0 | 7.39E+08 |
| P62258 | YWHAE | 4.5E+08 | 2.53E+08 |
| P09651 | HNRNPA1 | 1.6E+08 | 3.14E+08 |
| F2Z388 | RPL35 | 4.3E+07 | 5.35E+08 |
| P50990 | CCT8 | 4.9E+08 | 1.88E+08 |
| F8W7C6 | RPL10 | 9.6E+07 | 2.26E+08 |
| P13797 | PLS3 | 3.2E+08 | 3.47E+08 |
| P63241 | EIF5A | 4.8E+08 | 1.25E+08 |
| P30048 | PRDX3 | 0 | 6.78E+08 |
| P52565 | ARHGDIA | 5.7E+08 | 85275000 |
| P67809 | YBX1 | 1.6E+08 | 2.25E+08 |
| A0A3B3IUA2 | NHP2L1 | 5E+08 | 96659000 |
| P84077 | ARF1 | 4.2E+08 | 2.12E+08 |
| P00505 | GOT2 | 0 | 6.25E+08 |
| P00441 | SOD1 | 4.9E+08 | 1.07E+08 |
| P32119 | PRDX2 | 4.8E+08 | 85405000 |
| P09936 | UCHL1 | 5.7E+08 | 29213000 |
| P62820 | RAB1A | 7.3E+07 | 5.17E+08 |
| P13804 | ETFA | 0 | 5.92E+08 |
| Q9NZI8 | IGF2BP1 | 1.1E+08 | 1.75E+08 |
| P05386 | RPLP1 | 0 | 4.51E+08 |
| P04075 | ALDOA | 4.7E+08 | 73747000 |
| A6NLH6 | CNIH4 | 3.4E+07 | 5.49E+08 |
| E5RI99 | RPL30 | 1.3E+08 | 1.93E+08 |
| Q9Y3U8 | RPL36 | 3.4E+07 | 4.56E+08 |
| P68032 | ACTC1 | 1.6E+08 | 4.13E+08 |
| F8VNT9 | CD63 | 0 | 5.64E+08 |
| P49411 | TUFM | 0 | 5.7E+08 |
| Q13162 | PRDX4 | 1.6E+07 | 5.22E+08 |
| P30041 | PRDX6 | 4.4E+08 | 72288000 |
| M0R0F0 | RPS5 | 5.7E+07 | 2.2E+08 |
| Q96AG4 | LRRC59 | 0 | 5.08E+08 |
| P05141 | SLC25A5 | 0 | 5.03E+08 |
| P48643 | CCT5 | 3.8E+08 | 1.13E+08 |
| Q5W0H4 | TPT1 | 1.2E+08 | 2.53E+08 |
| Q9H9B4 | SFXN1 | 0 | 4.97E+08 |
| B4DDC6 | PTGES3 | 4.5E+08 | 38353000 |
| P78371 | CCT2 | 3.4E+08 | 1.37E+08 |
| R4GN19 | RPL36A | 0 | 0 |
| F5GWH5 | TMEM258 | 0 | 4.89E+08 |
| Q5H8X8 | UTS2 | 2E+07 | 4.47E+08 |
| Q00325 | SLC25A3 | 0 | 4.72E+08 |
| P12956 | XRCC6 | 2E+08 | 1.7E+08 |
| P49207 | RPL34 | 5.8E+07 | 1.64E+08 |
| P0DP25 | CALM2 | 2.7E+08 | 1.96E+08 |
| P11940 | PABPC1 | 1.1E+08 | 1.05E+08 |
| P26038 | MSN | 7.2E+07 | 3.88E+08 |
| Q99832 | CCT7 | 3.5E+08 | 95201000 |
| P04844 | RPN2 | 885160 | 4.52E+08 |
| G3V4C1 | HNRNPC | 5.7E+07 | 2.67E+08 |
| P61978 | HNRNPK | 1.8E+08 | 2.18E+08 |
| P50454 | SERPINH1 | 1.3E+07 | 4.33E+08 |
| Q04760 | GLO1 | 3.9E+08 | 51169000 |
| P09429 | HMGB1 | 1.8E+08 | 2.51E+08 |
| P62854 | RPS26 | 2.3E+07 | 3.26E+08 |
| P40227 | CCT6A | 3.3E+08 | 1.07E+08 |
| P51148 | RAB5C | 5E+07 | 3.98E+08 |
| P00558 | PGK1 | 3.7E+08 | 54466000 |
| Q00839 | HNRNPU | 1.5E+08 | 1.59E+08 |
| Q15366 | PCBP2 | 2.2E+08 | 1.18E+08 |
| Q14697 | GANAB | 1.9E+07 | 4.1E+08 |
| P24534 | EEF1B2 | 2.5E+08 | 1.32E+08 |
| D6R9P3 | HNRNPAB | 1E+08 | 1.75E+08 |
| P30086 | PEBP1 | 3.7E+08 | 41781000 |
| P04843 | RPN1 | 377820 | 4.17E+08 |
| P29692 | EEF1D | 1.4E+08 | 2.15E+08 |
| P35637 | FUS | 6.2E+07 | 1.72E+08 |
| Q99497 | PARK7 | 2.9E+08 | 1.19E+08 |
| O75390 | CS | 0 | 4.1E+08 |
| Q15907 | RAB11B | 7.6E+07 | 3.32E+08 |
| P06748 | NPM1 | 2E+08 | 1.69E+08 |
| P17987 | TCP1 | 3E+08 | 97293000 |
| B1AKQ8 | GNB1 | 1.4E+07 | 3.92E+08 |
| P00387 | CYB5R3 | 4203100 | 3.96E+08 |
| O43852 | CALU | 6999100 | 3.92E+08 |
| P13073 | COX4I1 | 0 | 4.03E+08 |
| P22234 | PAICS | 3.6E+08 | 24321000 |
| P12004 | PCNA | 2.9E+08 | 95135000 |
| P60468 | SEC61B | 0 | 3.82E+08 |
| P18669 | PGAM1 | 3.1E+08 | 65956000 |
| X6RJP6 | TAGLN2 | 2.5E+08 | 1.33E+08 |
| P51659 | HSD17B4 | 0 | 3.62E+08 |
| Q9NRW1 | RAB6B | 5.6E+07 | 3.18E+08 |
| A0A087WXM6 | RPL17 | 4.6E+07 | 1.95E+08 |
| P62834 | RAP1A | 1E+07 | 3.62E+08 |
| A0A0C4DFV9 | SET | 3.3E+08 | 26553000 |
| P62314 | SNRPD1 | 1.3E+08 | 2.25E+08 |
| P29401 | TKT | 2.8E+08 | 50115000 |
| P62942 | FKBP1A | 3E+08 | 50412000 |
| P49368 | CCT3 | 2.6E+08 | 97512000 |
| A0A087WY85 | UBE2D3 | 1.8E+08 | 1.64E+08 |
| P50991 | CCT4 | 2.5E+08 | 1.01E+08 |
| P29966 | MARCKS | 1.3E+08 | 2.21E+08 |
| P30040 | ERP29 | 1.3E+07 | 3.35E+08 |
| P04181 | OAT | 0 | 3.45E+08 |
| P53396 | ACLY | 2.8E+08 | 54912000 |
| O00571 | DDX3X | 7.4E+07 | 1.41E+08 |
| O60506 | SYNCRIP | 7.8E+07 | 1.01E+08 |
| P60866 | RPS20 | 7.5E+07 | 1.13E+08 |
| Q02790 | FKBP4 | 3.1E+08 | 28772000 |
| C9JFR7 | CYCS | 0 | 3.36E+08 |
| Q12906 | ILF3 | 6.8E+07 | 86094000 |
| A0A1B0GW44 | CTSD | 1567400 | 3.33E+08 |
| P23526 | AHCY | 3E+08 | 31462000 |
| Q12931 | TRAP1 | 0 | 3.32E+08 |
| E9PEX6 | DLD | 0 | 3.25E+08 |
| Q9P035 | HACD3 | 2176600 | 3.18E+08 |
| P55209 | NAP1L1 | 2.7E+08 | 56073000 |
| A0A0U1RRM4 | PTBP1 | 7E+07 | 1.07E+08 |
| H7BZJ3 | PDIA3 | 1.3E+07 | 2.94E+08 |
| P13010 | XRCC5 | 1.5E+08 | 75435000 |
| P36542 | ATP5C1 | 0 | 3.14E+08 |
| O96008 | TOMM40 | 0 | 3.13E+08 |
| O75531 | BANF1 | 8463600 | 2.9E+08 |
| Q15293 | RCN1 | 1.6E+07 | 2.95E+08 |
| P23246 | SFPQ | 3.8E+07 | 98726000 |
| M0R3D6 | RPL18A | 7.3E+07 | 92050000 |
| P40925 | MDH1 | 2.5E+08 | 41965000 |
| CON__P15636 |  | 1.1E+08 | 1.23E+08 |
| P49755 | TMED10 | 7968600 | 3.01E+08 |
| Q9NR28 | DIABLO | 0 | 3.01E+08 |
| B4DY09 | ILF2 | 6.1E+07 | 66182000 |
| Q99714 | HSD17B10 | 0 | 2.99E+08 |
| P46783 | RPS10 | 4.8E+07 | 1.63E+08 |
| P30049 | ATP5D | 0 | 2.98E+08 |
| P22314 | UBA1 | 2.6E+08 | 32880000 |
| P05023 | ATP1A1 | 1215200 | 2.95E+08 |
| F8W1R7 | MYL6 | 1.4E+08 | 1.54E+08 |
| E9PM31 | CD81 | 1.8E+07 | 2.76E+08 |
| P16615 | ATP2A2 | 743610 | 2.88E+08 |
| P06744 | GPI | 2.6E+08 | 15465000 |
| Q9H3N1 | TMX1 | 0 | 2.89E+08 |
| Q9Y230 | RUVBL2 | 2E+08 | 70724000 |
| A0A087X0X3 | HNRNPM | 8.7E+07 | 1.03E+08 |
| F8W1A4 | AK2 | 0 | 2.9E+08 |
| P34897 | SHMT2 | 0 | 2.89E+08 |
| Q14696 | MESDC2 | 5165600 | 2.84E+08 |
| P26583 | HMGB2 | 2.8E+07 | 2.44E+08 |
| Q9NPJ3 | ACOT13 | 0 | 2.88E+08 |
| P24752 | ACAT1 | 0 | 2.88E+08 |
| A0A1W2PQV2 | GCSH | 0 | 2.87E+08 |
| P62266 | RPS23 | 6.1E+07 | 77398000 |
| Q9HB71 | CACYBP | 2.1E+08 | 39760000 |
| J3KTA4 | DDX5 | 5.8E+07 | 86928000 |
| P22695 | UQCRC2 | 0 | 2.81E+08 |
| Q9Y3I0 | RTCB | 4.5E+07 | 74147000 |
| P31943 | HNRNPH1 | 9.1E+07 | 1.38E+08 |
| Q13283 | G3BP1 | 6.2E+07 | 1.34E+08 |
| Q7KZF4 | SND1 | 1.7E+07 | 1.65E+08 |
| P43487 | RANBP1 | 2E+08 | 63585000 |
| Q14974 | KPNB1 | 1.8E+08 | 90709000 |
| P61088 | UBE2N | 2.3E+08 | 33374000 |
| P52209 | PGD | 2.4E+08 | 18575000 |
| P42704 | LRPPRC | 0 | 2.69E+08 |
| Q15758 | SLC1A5 | 3911900 | 2.64E+08 |
| P20674 | COX5A | 3359200 | 2.65E+08 |
| P11586 | MTHFD1 | 1.4E+08 | 18997000 |
| Q8NBS9 | TXNDC5 | 6971700 | 2.56E+08 |
| P05455 | SSB | 2.3E+08 | 31974000 |
| P50502 | ST13 | 1.9E+08 | 72698000 |
| P09874 | PARP1 | 3.4E+07 | 1.82E+08 |
| P84103 | SRSF3 | 1.4E+07 | 1.83E+08 |
| Q07065 | CKAP4 | 0 | 2.65E+08 |
| Q13263 | TRIM28 | 2.4E+08 | 17496000 |
| Q9P1F3 | ABRACL | 2.6E+08 | 0 |
| Q9Y237 | PIN4 | 4.1E+07 | 1.1E+08 |
| P04264 | KRT1 | 8.2E+07 | 1.14E+08 |
| P55072 | VCP | 5.3E+07 | 2.01E+08 |
| Q13011 | ECH1 | 0 | 2.57E+08 |
| P22307 | SCP2 | 0 | 2.51E+08 |
| Q99613 | EIF3C | 7.5E+07 | 69566000 |
| Q15125 | EBP | 0 | 2.52E+08 |
| P34932 | HSPA4 | 2.2E+08 | 27847000 |
| C9JL85 | MTPN | 2.1E+08 | 44610000 |
| Q9UII2 | ATPIF1 | 0 | 2.53E+08 |
| P07355 | ANXA2 | 3978600 | 2.49E+08 |
| A0A286YF22 | PHGDH | 2.2E+08 | 33204000 |
| Q14257 | RCN2 | 4021200 | 2.52E+08 |
| P11177 | PDHB | 0 | 2.51E+08 |
| Q15233 | NONO | 3.7E+07 | 55448000 |
| Q16576 | RBBP7 | 2E+08 | 46074000 |
| P39656 | DDOST | 0 | 2.4E+08 |
| O95433 | AHSA1 | 2.4E+08 | 7015200 |
| P49321 | NASP | 2.2E+08 | 16957000 |
| Q9Y277 | VDAC3 | 0 | 2.41E+08 |
| Q9NVJ2 | ARL8B | 8362100 | 2.3E+08 |
| H0Y4R1 | IMPDH2 | 2.2E+08 | 18430000 |
| Q96AY3 | FKBP10 | 3132400 | 2.32E+08 |
| H0YLA2 | SRP14 | 6.4E+07 | 1.33E+08 |
| Q13838 | DDX39B | 1.1E+08 | 65718000 |
| A0A0A0MSE2 | HADH | 0 | 2.31E+08 |
| P49006 | MARCKSL1 | 4.2E+07 | 1.87E+08 |
| Q01130 | SRSF2 | 9.5E+07 | 1.3E+08 |
| P61586 | RHOA | 5.3E+07 | 1.74E+08 |
| O14818 | PSMA7 | 2E+08 | 20240000 |
| B5MCS2 | SETD7 | 0 | 2.21E+08 |
| Q14011 | CIRBP | 4.6E+07 | 67809000 |
| Q49AN9 | SNRPG | 9.8E+07 | 1.05E+08 |
| P35579 | MYH9 | 1.5E+08 | 72258000 |
| P28070 | PSMB4 | 2E+08 | 10493000 |
| Q9Y265 | RUVBL1 | 1.5E+08 | 47709000 |
| P48047 | ATP5O | 0 | 2.16E+08 |
| Q01581 | HMGCS1 | 2E+08 | 15410000 |
| K7ELC2 | RPS15 | 5.7E+07 | 16145000 |
| H0YJW7 | SLIRP | 0 | 2.13E+08 |
| P50395 | GDI2 | 1.9E+08 | 19364000 |
| Q15181 | PPA1 | 1.8E+08 | 29536000 |
| Q53GQ0 | HSD17B12 | 0 | 2.12E+08 |
| Q14444 | CAPRIN1 | 4.7E+07 | 92719000 |
| P61009 | SPCS3 | 0 | 2.05E+08 |
| A2A274 | ACO2 | 0 | 2.08E+08 |
| D3YTB1 | RPL32 | 1.4E+07 | 1.09E+08 |
| B4DR61 | SEC61A1 | 0 | 2.06E+08 |
| A0A5F9YFS9 | SPCS1 | 0 | 2.07E+08 |
| P49327 | FASN | 1.8E+08 | 20949000 |
| Q02978 | SLC25A11 | 0 | 2.07E+08 |
| P08559 | PDHA1 | 0 | 2.06E+08 |
| Q9Y4L1 | HYOU1 | 3895700 | 2E+08 |
| P62495 | ETF1 | 1E+08 | 71696000 |
| P20042 | EIF2S2 | 7.9E+07 | 91589000 |
| Q15363 | TMED2 | 8785700 | 1.95E+08 |
| P52907 | CAPZA1 | 1.2E+08 | 78936000 |
| P04179 | SOD2 | 0 | 2.03E+08 |
| P47813 | EIF1AX | 1.3E+08 | 42226000 |
| H0YN26 | ANP32A | 1.5E+08 | 33406000 |
| Q9P0L0 | VAPA | 1268600 | 1.99E+08 |
| P55884 | EIF3B | 7.4E+07 | 45193000 |
| P10606 | COX5B | 0 | 2E+08 |
| O95831 | AIFM1 | 0 | 2E+08 |
| B1AK88 | CAPZB | 1.1E+08 | 77581000 |
| P63104 | YWHAZ | 1.3E+08 | 65494000 |
| P07741 | APRT | 1.8E+08 | 21610000 |
| P08133 | ANXA6 | 2102000 | 1.96E+08 |
| A0A3B3IRT8 | SSR1 | 0 | 1.98E+08 |
| Q9NSD9 | FARSB | 3.2E+07 | 24193000 |
| Q16891 | IMMT | 0 | 1.96E+08 |
| Q3ZCQ8 | TIMM50 | 0 | 1.96E+08 |
| O95881 | TXNDC12 | 2767900 | 1.91E+08 |
| Q9Y5M8 | SRPRB | 0 | 1.92E+08 |
| Q15836 | VAMP3 | 0 | 1.9E+08 |
| Q9Y3F4 | STRAP | 1.2E+08 | 45035000 |
| P49419 | ALDH7A1 | 6.1E+07 | 1.28E+08 |
| Q08211 | DHX9 | 3.4E+07 | 56806000 |
| P28066 | PSMA5 | 1.6E+08 | 21214000 |
| P05091 | ALDH2 | 0 | 1.87E+08 |
| O43707 | ACTN4 | 5.9E+07 | 1.27E+08 |
| O00264 | PGRMC1 | 0 | 1.84E+08 |
| P48735 | IDH2 | 7322200 | 1.75E+08 |
| O60869 | EDF1 | 1.1E+07 | 42122000 |
| P55060 | CSE1L | 1.5E+08 | 19759000 |
| A0A087WVQ6 | CLTC | 9.1E+07 | 87789000 |
| P50402 | EMD | 0 | 1.81E+08 |
| P54727 | RAD23B | 1.5E+08 | 31959000 |
| M0QXF7 | MYDGF | 6263800 | 1.75E+08 |
| P21912 | SDHB | 0 | 1.81E+08 |
| E9PES6 | HMGB3 | 2.7E+07 | 1.5E+08 |
| P61019 | RAB2A | 2192800 | 1.79E+08 |
| P25789 | PSMA4 | 1.6E+08 | 14969000 |
| H0YFD6 | HADHA | 0 | 1.79E+08 |
| M0QXH0 | TXN2 | 0 | 1.78E+08 |
| O00303 | EIF3F | 7.6E+07 | 46181000 |
| P61026 | RAB10 | 1E+07 | 1.67E+08 |
| P51571 | SSR4 | 0 | 1.77E+08 |
| O00299 | CLIC1 | 1.3E+08 | 48379000 |
| Q9BVK6 | TMED9 | 3715800 | 1.69E+08 |
| P05787 | KRT8 | 1.3E+08 | 47179000 |
| P31948 | STIP1 | 1.4E+08 | 33068000 |

| eEF1a |
| --- |
| eEF2 |
| other factors |
| ribosomal proteins |
| ER-resident proteins |
| mitochondrial proteins |

# Supplementary Video Captions

**Supplementary Video 1. The elongation cycle**. Experimentally observed elongation intermediates are arranged in the model of the human elongation cycle.

**Supplementary Video 2**. **Conformational changes of eEF1a on ribosomes**. Morph illustrating the conformational change of eEF1a from the compact conformation (decoding-recognition state, 5LZS) to the extended conformation (classical-pre+ state).
