# Supplementary material for: Visualization of translation and protein biogenesis at the ER membrane
Source: Nature. 2023 Jan 25;614(7946):160–7. doi: 10.1038/s41586-022-05638-5 (PMC9892003; doi:10.1038/s41586-022-05638-5)
Supplement: Supplementary file 2 — Reporting Summary [file 41586_2022_5638_MOESM2_ESM.pdf]

nature portfolio

Corresponding author(s): Friedrich Forster

Last updated by author(s): Nov 22, 2022

Reporting Summary

Nature Portfolio wishes to improve the reproducibility of the work that we publish. This form provides structure for consistency and transparency in reporting. For further information on Nature Portfolio policies, see our Editorial Policies and the Editorial Policy Checklist.

Statistics

For all statistical analyses, confirm that the following items are present in the figure legend, table legend, main text, or Methods section.

n/a	Confirmed	
☐	☒	The exact sample size (n) for each experimental group/condition, given as a discrete number and unit of measurement
☐	☒	A statement on whether measurements were taken from distinct samples or whether the same sample was measured repeatedly
☐	☒	The statistical test(s) used AND whether they are one- or two-sided<br>*Only common tests should be described solely by name; describe more complex techniques in the Methods section.*
☐	☒	A description of all covariates tested
☒	☐	A description of any assumptions or corrections, such as tests of normality and adjustment for multiple comparisons
☐	☒	A full description of the statistical parameters including central tendency (e.g. means) or other basic estimates (e.g. regression coefficient) AND variation (e.g. standard deviation) or associated estimates of uncertainty (e.g. confidence intervals)
☐	☒	For null hypothesis testing, the test statistic (e.g. F, t, r) with confidence intervals, effect sizes, degrees of freedom and P value noted<br>*Give P values as exact values whenever suitable.*
☒	☐	For Bayesian analysis, information on the choice of priors and Markov chain Monte Carlo settings
☒	☐	For hierarchical and complex designs, identification of the appropriate level for tests and full reporting of outcomes
☒	☐	Estimates of effect sizes (e.g. Cohen's d, Pearson's r), indicating how they were calculated

Our web collection on statistics for biologists contains articles on many of the points above.

Software and code

Policy information about availability of computer code

Data collection	Tilt series were acquired using SerialEM 3.8 and GMS 2.3. Single particle cryo-EM data were acquired using EPU 3.
Data analysis	For subtomogram averaging and classification we used the following software: Warp 1.0.9, M 1.0.9, Relion 3.1.1, PyTOM 0.994, IMOD 4.10.25. For segmentation we used Eman2 2.91 and for visualization Chimera 1.14.0, ChimeraX 1.3.0. To analyze polysomes we used an in-house developed python package (https://github.com/McHaillet/polysome-stats) that made use of mclogit 0.9.4.2, R 3.6.1, Python 3.8.11, Numpy 1.20.3 and Scipy 1.7.1.<br>For cryo-EM single particle analysis we used Relion 3.1.1. For atomic model building and assessment we used Isolde 1.0b5, Phenix 1.20.1, Coot 0.9.8.2, Imodfit 1.51, Molprobity 4.5.1, and findMySequence (https://gitlab.com/gchojnowski/findmysequence, not versioned), Mass-spec analysis was performed using MaxQuant 2.0.1.0.

For manuscripts utilizing custom algorithms or software that are central to the research but not yet described in published literature, software must be made available to editors and reviewers. We strongly encourage code deposition in a community repository (e.g. GitHub). See the Nature Portfolio guidelines for submitting code & software for further information.

Data

Policy information about availability of data

All manuscripts must include a data availability statement. This statement should provide the following information, where applicable:

- Accession codes, unique identifiers, or web links for publicly available datasets
- A description of any restrictions on data availability
- For clinical datasets or third party data, please ensure that the statement adheres to our policy

Data generated in this study are available in the main article, supplementary materials or in public repositories: nos. EMD-15870, EMD-15871, EMD-15872, EMD-15873, EMD-15874, EMD-15875, EMD-15876, EMD-15877, EMD-15878, EMD-15879, EMD-15880, EMD-15884, EMD-15885, EMD-15886, EMD-15887, EMD-15888, EMD-15889, EMD-15890, EMD-15891, EMD-15892, EMD-15893 of EMDB (www.ebi.ac.uk/emdb) and PDB-8B6Z, PDB-8B6L of PDB (www.rcsb.org). The mass spectrometry proteomics data have been deposited to the ProteomeXchange Consortium via the PRIDE68 partner repository with the dataset identifier PXD035475.
In addition, we made use of a previously published atomic models from the PDB (accession codes 5AJO, 4CXG, 4UJE, 6Y0G, 6Y57, 6GZ5, 6Z6L, 6Z6M, 5LZS, 4C0S, 5LZT, 5IZK, 6O85, 5LZZ, 6GZ3, 6GZ4, 6GZ5, 6SXO, 1BN5, 6W6L, 6ENY) and the AlphaFold Protein Structure Database (AF-O00178, AF-P30101). Moreover, we used the following EM densities from the EMDB for analyses: EMDB-2904, EMDB-2908.

Field-specific reporting

Please select the one below that is the best fit for your research. If you are not sure, read the appropriate sections before making your selection.

☒ Life sciences ☐ Behavioural & social sciences ☐ Ecological, evolutionary & environmental sciences

For a reference copy of the document with all sections, see nature.com/documents/nr-reporting-summary-flat.pdf

Life sciences study design

All studies must disclose on these points even when the disclosure is negative.

Sample size	No sample size calculation was performed. Cryo-EM structures were determined from a single sample based on 869 tilt series containing 134,350 particles. Target for cryo-ET subtomogram analysis was at least 100,000 particles, surpassing previous analysis by one order of magnitude allowing for better classification. The rationale for 100,000 particles was that a class representing 1% of intermediates would contain 1,000 particles, which is sufficient to obtain sub-nanometer resolution.<br>Classification was repeated for 2 independent samples (see Replication). MS analysis was performed in technical replicates from one sample.
Data exclusions	Tiltseries from thick samples were excluded due to poor signal-to-noise ratio.
Replication	Microsome preparation and translocon analysis has been repeated twice from different cell batches with lower acquisition statistics (Extended Figures 4F). The two replicates comprised 31 tomograms (6,101 particles) and 69 tomograms (3,836 particles), respectively. The same translation intermediates were detected in replicates.
Randomization	Randomization was not performed for cryo-EM analysis as there is nothing to randomize.
Blinding	Blinding is not technically feasible for structure determination.

Reporting for specific materials, systems and methods

We require information from authors about some types of materials, experimental systems and methods used in many studies. Here, indicate whether each material, system or method listed is relevant to your study. If you are not sure if a list item applies to your research, read the appropriate section before selecting a response.

Materials & experimental systems

n/a	Involved in the study	
☐	☒	Antibodies
☐	☒	Eukaryotic cell lines
☒	☐	Palaeontology and archaeology
☒	☐	Animals and other organisms
☒	☐	Human research participants
☒	☐	Clinical data
☒	☐	Dual use research of concern

Methods

n/a	Involved in the study	
☒	☐	ChIP-seq
☒	☐	Flow cytometry
☒	☐	MRI-based neuroimaging

Antibodies

Antibodies used	anti-Sec61alpha (Abcam, ab15575, polyclonal; dilution: 1:1000), anti-SSR3 (Sigma Aldrich, hpa014906, polyclonal; dilution: 1:1000), anti-CCDC47 (Abcam, ab241608, polyclonal; dilution: 1:1000).
Validation	anti-Sec61alpha: validated by WB of murine dendritic cells (DOI: 10.4049/jimmunol.1302312), anti-SSR3: validated by WB of human A549 cells (DOI: 10.1126/sciadv.abc6364), anti-CCDC47: validated by WB of HEK-293T whole lysate (manufacturer)

Eukaryotic cell lines

Policy information about cell lines

Cell line source(s)	FreeStyle 293-F cells (ThermoFisher Scientific, R79007), U2OS and HeLa cell originated from ATCC (CVCL_0042 and CVCL_0030 in Cellosaurus.org, respectively)
Authentication	Cell lines were not authenticated
Mycoplasma contamination	Cell lines tested negative for Mycoplasma contamination
Commonly misidentified lines (See ICLAC register)	No commonly misidentified cell lines were used
